# Multivariate variance-components analysis of longitudinal blood pressure measurements from the Framingham Heart Study

**DOI:** 10.1186/1471-2156-4-S1-S55

**Published:** 2003-12-31

**Authors:** Peter Kraft, Lara Bauman, Jin Ying Yuan, Steve Horvath

**Affiliations:** 1 Department of Epidemiology, Harvard School of Public Health, Boston, MA, USA; 2 Department of Biomathematics, University of California at Los Angeles, Los Angeles, California, USA; 3 Department of Human Genetics, University of California at Los Angeles, Los Angeles, California, USA; 4 Department of Biostatistics, University of California at Los Angeles, Los Angeles, California, USA

## Abstract

Multivariate variance-components analysis provides several advantages over univariate analysis when studying correlated traits. It can test for pleiotropy or (in the longitudinal context) gene × age interaction. It can also have more power than univariate analyses to detect a quantitative trait locus influencing several traits. We apply multivariate variance components to longitudinal systolic blood pressure data from the Framingham Heart Study. We find evidence for a polygenic influence on blood pressure (heritabilities at different ages range from 27% to 38%). Tests based on a factor-analytic parameterization of the polygenic variance find significant (*p *< 2 × 10^-3^) evidence that different genes affect blood pressure at different ages. Still, estimates for the proportion of polygenic variance due to shared genes ran as high as 85% for some trait pairs. Univariate and multivariate linkage analyses replicate previous linkage results on chromosome 17 (maximum LOD scores of 2.2 and 2.4, respectively). In this study, multivariate analysis provides no increase in power; this is likely due to the strong positive correlation in systolic blood pressure measured at different ages.

## Background

High blood pressure is a complex disorder that results from environmental and genetic factors and their interactions. Levy et al. [1] found evidence for a gene influencing blood pressure on chromosome 17 using data from the Framingham Heart Study. However, this study analyzed average blood pressure over a 50-year period (ages 25 to 75), and may not have taken full advantage of the longitudinal nature of the Framingham study. Blood pressure increases with age; there may be genes that influence the rate of this increase. Similarly, there may be genes that influence blood pressure only at early or late ages. For example, a segregation analysis by Pérusse et al. [2] suggested that blood pressure is influenced by a major gene with age-dependent effects. Animal studies have also found that different genes can influence a trait at different ages [3]. Taking lifetime averages may mask such effects.

de Andrade et al. [4] recently analyzed longitudinal quantitative trait data using a multivariate variance components approach. This approach can be more powerful for correlated traits than a univariate approach [5]. It can also test for gene × time interaction, polygenic pleiotropy – defined in the longitudinal context as a trait being determined by the same set of genes at distinct time points – and distinguish between major gene pleiotropy and co-incident linkage [6-8].

de Andrade et al. [4] defined traits by calendar time. Thus, for a cohort study with age-staggered entry, each measurement will have been taken at approximately the same calendar time for all subjects, but at different biological ages. In the context of the Framingham study we propose to define traits by biological age, so as to distinguish genes involved in determining high blood pressure at young or old ages as opposed to uncovering a gene × calendar time (environment) interaction.

We apply univariate and multivariate variance components to systolic blood pressure measurements on subjects from the Framingham Heart Study taken in four different age ranges. We consider models with a polygenic component and both a polygenic and major gene component. We describe and apply a test of the null hypothesis of complete pleiotropy versus the alternative of incomplete pleiotropy based on a factor-analytic parameterization of the polygenic variance component. We reject the null hypothesis of complete pleiotropy, suggesting a different set of genes influence systolic blood pressure at different ages. We find linkage signals on chromosome 17 consistent with the earlier report of Levy et al. [1].

## Methods

### Subjects, trait definitions, marker data

Phenotype data were available for 2885 subjects from 330 pedigrees (with a total of 4692 members) from the Framingham Heart Study. The data included age, systolic blood pressure (mm Hg), hypertension treatment (yes/no), sex, height, and weight measured at 2- to 4-year intervals. Data were not available on every subject at the same set of ages because of staggered entry, drop-out, and intermittent missing data.

To ensure that we had phenotype data on comparable ages for as many subjects as possible, we averaged systolic blood pressure (SBP) and body mass index (BMI) over any measurements taken during four age intervals: younger than 35 years; between 35 and 50; between 50 and 55; and older than 65. On average, 1.6, 5.3, 6.3, and 5.9 SBP measurements were available on original cohort members in these four age intervals, respectively. The corresponding numbers for the offspring cohort were 1.8, 2.2, 2.2, and 1.6 (lower due to the longer interval between exams). Interval-specific hypertension treatment phenotypes were defined to be "yes" if the subject received any hypertension treatment during the interval and "no" otherwise.

We adjusted SBP for the effect of hypertension treatment using a procedure similar to that outlined by Levy et al. [1] for each age interval separately. The adjusted SBP values for each age interval were then regressed on sex and BMI. We used the residuals from this regression as quantitative trait(s) in (multivariate) variance components analyses described below.

Marker genotype data were available on 1702 subjects. We used data on 16 markers along chromosome 17; the markers were roughly equidistant, one per 10 cM.

### Variance components for multivariate traits

For each subject *j *= 1,...,*J*_*i*_, in family *i *= 1,...,*I*, let **Y**_*ij *_be the *p*-dimensional vector of SBP residuals. We take *p *= 1, 2, or 4 for (respectively) the four age intervals considered separately, the two intervals 35–50 and 50–65 considered simultaneously, and all four intervals considered simultaneously.

We use the variance components model **Y**_*ij *_= **μ **+ **X β **+ **a**_*ij *_+ **g**_*ij *_+ **e**_*ij *_[8-10]. Here μ and β are fixed effects, and **X **is a matrix of (possibly age-specific) covariates. For these analyses we fit no fixed effects as we regressed on relevant covariates when creating the age-interval-specific trait data. The **a**_*ij*_, **g**_*ij*_, and **e**_*ij *_terms are multivariate normal random effects with mean **0**. Here **a**_*ij *_is an additive polygenic effect, **g**_*ij *_is the additive effect due to a specific locus, and **e**_*ij *_is individual-specific error.

Writing **Y **= (**Y**_11_' **Y**_12_' ... **Y**_*IJi*_')' as the *p *(Σ_*i *_*J*_*i*_) × 1 = *p n *× 1 concatenated vector of all subjects' trait vectors,

Cov(**Y**,**Y**) = **K****A **+ **Π****G **+ **I ****E**,

where K is the *n *× *n *matrix of subjects' kinship coefficients; Π is the *n *× *n *matrix of identity-by-descent (IBD) sharing probabilities for all possible pairs of subjects (calculated at a given location using marker data); **I **is the *n *× *n *identity matrix; **A **is Cov(**a**_*ij*_,**a**_*ij*_) = {σ_*akl*_^2^}, with *k *and *l *indexing age intervals; **G **is Cov(**g**_*ij*_,**g**_*ij*_) = {σ_*gkl*_^2^}; and **E **is Cov(**e**_*ij*_,**e**_*ij*_) = {σ_*ekl*_^2^}. Particular parametric forms for **A**, **G **or **E **– such as autoregressive or exponential decay – can be adopted [6,7]. In this case, since *p *is small, we used the general form for **E **(which can be parameterized in terms of the variances σ_*ekk*_^2 ^≥ 0 and correlations -1 ≤ ρ_*kl *_≤ 1, *k*, *l *= 1,...,*p*).

Polygenic heritabilities can be estimated by fitting the reduced model with **g**_*ij *_≡ **0**. To test the null hypothesis of complete polygenic pleiotropy, we first fit the constrained model ρ_*kl *_≡ 1 by writing **A **= Δ Δ', with Δ' = (δ_1_,...,δ_*p*_). This model is compared to the general model (with the *q *= *p *(*p *- 1)/2 correlation parameters allowed to range between -1 and 1) via a likelihood ratio test. Asymptotically, this test statistic is distributed as the mixture of υ_*r*_^2 ^variables *r *= 0,...,*q*, with mixing probabilities Binom(*q*,*r*) 2^-*q *^[11]. The proportion of polygenic variance for two traits due to shared genes can be estimated as ρ_*kl*_^2 ^or (σ_*akl*_^2^/σ_*akk*σ*all*_)^2 ^[6].

LOD scores for linkage can be calculated by taking the log_10 _of the likelihood ratio of the model, which estimates **G **to the polygenic model with **g**_*ij *_≡ **0**. We use the general form for **A **and **G**. In principle, models with varying constraints on these variance matrices could be compared via Aikake's information criterion.

We used SIMWALK2 [12] to calculate IBD sharing and Fisher [13] to calculate maximum likelihood estimates for the variance components parameters. Fisher allows the user to specify that the polygenic (or major gene) variance have the structure **A **= Λ Λ ', with



Here Λ_1 _is a lower triangular *s *× *s *matrix (*s *<*p*) and Λ_2 _is a general (*p *- *s*) × *s *matrix. This amounts to parameterizing **A **in terms of *s *independent factors. Taking *s *= 1 leads to the parameterization **A **= Δ Δ' described above; taking *s *= *p *leads to a Cholesky decomposition of the general form for **A**.

## Results

Both the univariate and multivariate analyses find evidence for a polygenic component to SBP. The estimates of polygenic variance for residual SBP from all models are more than 3 standard errors away from 0 (Tables 1,2,3). Univariate heritabilities range from 27% to 37%. The multivariate analyses show strong correlation between the polygenic components for different age ranges; correlations range from 0.47 to 0.92 (Table 4).

Tests for incomplete pleiotropy are significant, however (test statistics of 8.4 and 21.8 with *p*-values of 2 × 10^-3 ^and 2 × 10^-4 ^for the two- and four-trait analyses, respectively). This suggests that the set of genes involved in regulating blood pressure differs with age. According to parameter estimates from the unconstrained four-trait analysis (Table 3), the proportion of variance due to shared genes for SBP between age intervals 35–50 and 50–65 is about 82%, but the proportion due to shared genes between age intervals 0–35 and 65+ is 22%.

Univariate LOD scores for the 50–65 age range achieve a peak of 2.2 on chromosome 17 at approximately 80 cM. Bivariate lod scores considering the residuals from the 35–50 and 50–65 age ranges peak just over 2.4 at the same location. This position corresponds roughly to that found by Levy et al. [1] with a lod score of 4.7.

## Discussion

Our results suggest that different sets of genes regulate blood pressure at different ages. Havill and Mahaney [14] find evidence for incomplete pleiotropy in the polygenic component of SBP in the fourth and sixth decades of life, although their estimates of the proportion of polygenic variance due to shared genes are lower than ours for comparable age ranges. These differences may be due to different sample sizes or trait definitions. Furthermore, Havill and Mahaney restrict their analysis to Framingham subjects measured at both age intervals; we did not.

We also find linkage signals on chromosome 17 consistent with the earlier report [1], although with smaller LOD scores. This reduction may be due to smaller sample size or differences in trait definition. Our analyses also do not take other known risk factors for high blood pressure such as smoking or cholesterol into account, although fixed effects for these factors can be included in the variance components model. Similarly, fixed effects for birth cohort or calendar time could be included, perhaps to capture differences in unmeasured risk factors. However, fitting age, birth cohort, and calendar time effects simultaneously could lead to problems with model identifiability [15].

The small increase in multivariate LOD scores over univariate LOD scores likely reflects the fact that when traits are strongly positively correlated, univariate analyses are more powerful [5]. This may also be why the approach of averaging SBP measures taken by Levy et al. [1] performs better than multivariate analysis in this case.

In contrast to our approach, which defines traits based on Framingham subjects' biological age, de Andrade and Olswold [16] define traits based on calendar time (exam number). They fail to replicate the linkage signal on chromosome 17. This is consistent with the results of Mathias et al. [17], who found that age-matched analyses of SBP from the Framingham Heart Study produced higher estimates of heritability than calendar-year-matched analyses. This suggests care should be taken when defining traits in a longitudinal analysis, depending on whether gene × age interaction or gene × calendar time (environment) interaction is more relevant.

A general drawback to the multivariate variance components approach sketched here is that missing observations on subjects with incomplete data are assumed to be missing at random [6] and hence ignorable. However, non-ignorable missingness is a concern in this case, where early withdrawal from study is likely correlated with elevated blood pressure (and hence any genes associated with high blood pressure). Multiple imputation methods can be applied in the case of non-ignorable missing data, as discussed by Allison [18] and several contributions to GAW13 [19,20].

## Figures and Tables

**Figure 1:**
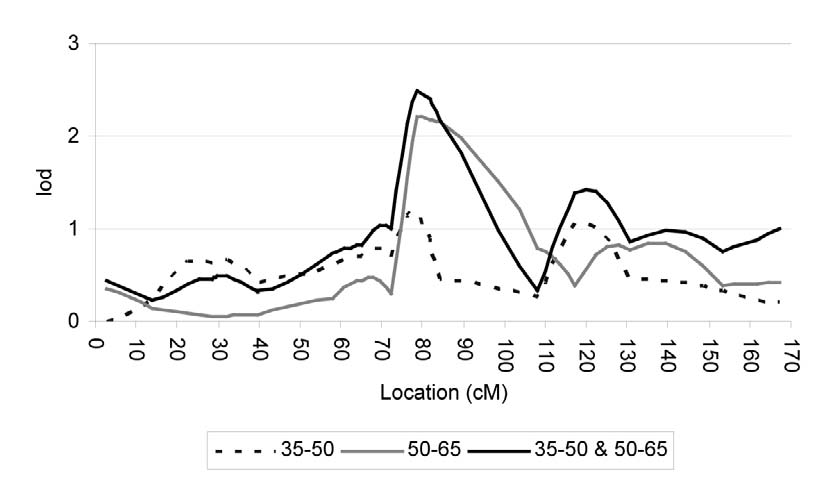
QTL LOD scores for SBP residuals, chromosome 17.

**Table 1:** Univariate estimates of covariance parameters (and their standard errors) from the polygenic model

Age interval	Polygenic σ_a_^2^	Error σ_e_^2^	*n*
< 35 years	34.99 (7.59)	95.13 (7.38)	1277
35–50 years	63.74 (7.45)	128.13 (6.76)	2411
50–65 years	109.44 (14.76)	214.57 (13.43)	2030
> 65 years	317.36 (67.16)	535.43 (63.14)	1133

**Table 2:** Multivariate^A ^(*p *= 2) estimates of covariance parameters (and their standard errors) from the polygenic model

Model	Estimate
*Constrained model (ρ = 1)*	
Polygenic variance **A**	
Error variance **E**	
Log-likelihood	-13,862
*Unconstrained model*	
Polygenic variance **A**	
Error variance **E**	
Log-likelihood	-13,858

^A^Bivariate trait consisting of measurements from the intervals 35–50 and 50–65 years.

**Table 3:** Multivariate^A ^(*p *= 4) estimates of covariance parameters (and their standard errors) from the polygenic model.

Model	Estimate
*Constrained model (ρ = 1)*	
Polygenic variance **A**	
Error variance **E**	
Log-likelihood	-21,462
*Unconstrained model*	
Polygenic variance **A**	
Error variance **E**	
Log-likelihood	-21,451

^A^Multivariate trait consisting of measurements from all four age intervals.

**Table 4:** Estimates of heritabilities from the polygenic model

	Heritability			Polygenic Correlation^A^			
		
Age interval	*p *= 1	*p *= 2	*p *= 4	< 35	35–50	50–65	>65
< 35 years	27%	NA	31%				
35–50 years	33%	36%	38%	0.92			
50–65 years	34%	32%	33%	0.76	0.90		
> 65 years	37%	NA	24%	0.47	0.64	0.70	

^A^Based on the multivariate model with *p *= 4.
